# Wnt signaling polarizes cortical actin polymerization to increase daughter cell asymmetry

**DOI:** 10.1038/s41421-022-00376-4

**Published:** 2022-03-01

**Authors:** Yongping Chai, Dong Tian, Zhiwen Zhu, Yuxiang Jiang, Shanjin Huang, Dou Wu, Guangshuo Ou, Wei Li

**Affiliations:** 1 Tsinghua-Peking Center for Life Sciences, Beijing Frontier Research Center for Biological Structure, McGovern Institute for Brain Research, School of Life Sciences and MOE Key Laboratory for Protein Science, Tsinghua University, Beijing, China; 2 School of Life Sciences, Tsinghua University, Beijing, China; 3 School of Medicine, Tsinghua University, Beijing, China

**Keywords:** Actin, Mitosis

## Abstract

Asymmetric positioning of the mitotic spindle contributes to the generation of two daughter cells with distinct sizes and fates. Here, we investigated an asymmetric division in the *Caenorhabditis elegans* Q neuroblast lineage. In this division, beginning with an asymmetrically positioned spindle, the daughter-cell size differences continuously increased during cytokinesis, and the smaller daughter cell in the posterior eventually underwent apoptosis. We found that Arp2/3-dependent F-actin assembled in the anterior but not posterior cortex during division, suggesting that asymmetric expansion forces generated by actin polymerization may enlarge the anterior daughter cell. Consistent with this, inhibition of cortical actin polymerization or artificially equalizing actin assembly led to symmetric cell division. Furthermore, disruption of the Wnt gradient or its downstream components impaired asymmetric cortical actin assembly and caused symmetric division. Our results show that Wnt signaling establishes daughter cell asymmetry by polarizing cortical actin polymerization in a dividing cell.

## Introduction

Asymmetric cell divisions (ACDs) are essential for organismal development and tissue homeostasis. Metazoan development involves the specification of various cell types from a single fertilized egg. Cell-type diversification can be achieved by ACDs that generate two daughter cells with identical genetic materials but distinct developmental potentials^1–6^. During ACDs, cell-intrinsic mechanisms that facilitate binary cell fate decisions entail the polarized segregation of cell fate determinants on the cell cortex, the asymmetric partition of RNA species, biased distribution of intracellular organelles, and unequal segregation of damaged proteins or protein aggregates^1,2^. In addition to asymmetric segregation of cellular composition, ACDs generate two daughter cells of distinct dimensions.

Daughter-cell-size asymmetry can result from asymmetric cleavage furrow positioning or unequal myosin-based contractility in the cortex^3,4^. The best-characterized example of asymmetric furrow position is the *Caenorhabditis elegans* (*C. elegans*) zygote. During the ACD of the one-cell stage embryo, dynein-mediated pulling forces displace the spindle toward the posterior pole at the end of the metaphase. Accordingly, the cleavage furrow forms at the center of the spindle but is shifted toward the rear, creating a large anterior daughter and a small posterior one^5^. The *C. elegans* Q.a and *Drosophila* neuroblast use a similar myosin asymmetry-based mechanism to generate two daughter cells of different sizes. During their ACDs, spindles are aligned in the middle of the dividing cells without any apparent displacement. At cytokinesis, non-muscle myosin II assembles and contracts at the contractile ring as all the other cells; however, myosin II becomes enriched asymmetrically in the cell cortex during anaphase. Polarized myosin-II assembly generates unequal cortical contractility to shrink one hemisphere, decreasing the daughter-cell size from this pole but increasing the daughter-cell dimension from the opposite, producing two differently sized daughter cells^3,4^.

**Fig. 1:**
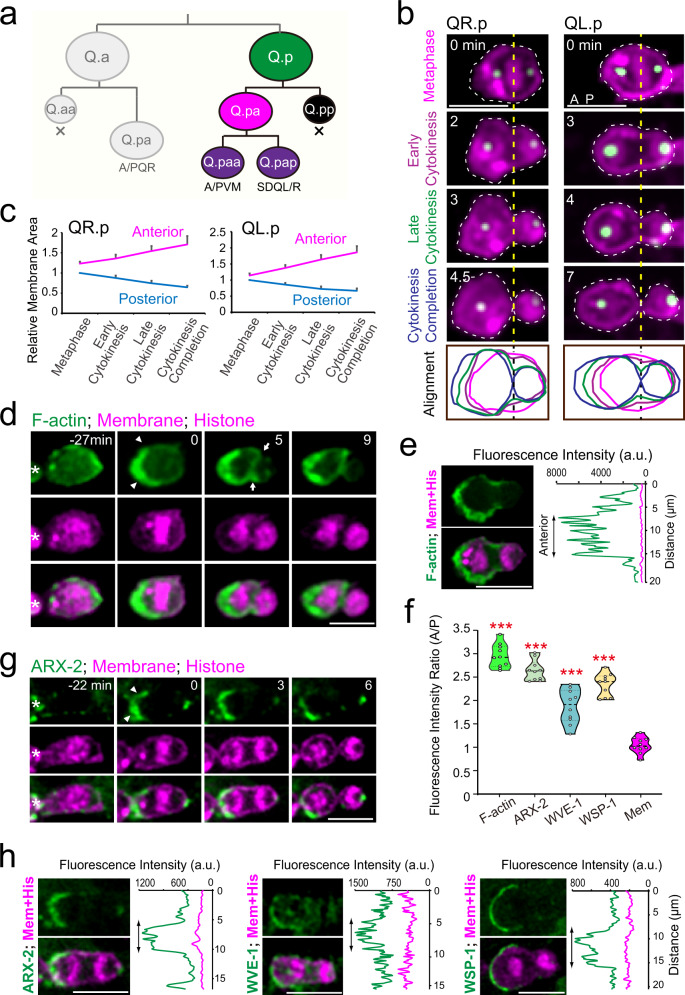
Asymmetric plasma membrane expansion and cortical actin polymerization during Q.p cytokinesis. **a** Q.p neuroblast on the left side (QL.p) or the right side (QR.p) of the animal generates two neurons (PVM and SDQL from QL, or AVM and SDQR from QR) and an apoptotic cell (X). **b** Time-lapse fluorescence images show the plasma membrane dynamics of Q.p cells during cytokinesis with an aligned cell periphery at the bottom. Magenta: mCherry-tagged with myristoylation (Myri) signal for marking the plasma membrane and mCherry-tagged histone H2B for chromosomes; green for centrosomes. Dotted yellow and black lines indicate the equatorial plate and contractile ring position. Dotted white lines show cell peripheries in images that were aligned at the bottom panel and color-coded according to their cytokinesis stages. **c** Quantification of the relative anterior and posterior (normalized to the metaphase posterior) plasma membrane area during QR.p (*n* = 9) and QL.p (*n* = 10) cytokinesis. **d** GFP::moesinABD (F-actin) dynamics during QR.p division. Arrowheads indicate the cortical fluorescence enrichment; arrows indicate fluorescence enrichment on the contractile ring; asterisks indicate neighboring Q cells. **e** Images of GFP-tagged moesinABD (F-actin), and mCherry-tagged plasma membrane and histone in cytokinetic QR.p, and the line-scan intensity plots of F-actin (green) with Myri-mCherry (magenta) signal around the QR.p periphery. The trace begins from the posterior and moves counterclockwise along the cell periphery to the anterior and then back to the posterior (Refer to Supplementary Fig. S1a). **f** Violin plots with all data points of the fluorescence intensity ratio of indicated proteins and the plasma membrane (Mem) between the anterior and posterior of the cytokinetic QR.p (*n* = 10 cells in each group). **g** Fluorescence images of ARX-2::GFP dynamics during QR.p division. Arrowheads indicate the cortical fluorescence enrichment; asterisks indicate neighboring Q cells. **h** Images of GFP-tagged ARX-2, WVE-1 and WSP-1, and mCherry-tagged plasma membrane and histone in cytokinetic QR.p, and their line-scan intensity plots (green) with Myri-mCherry (magenta) signal around the periphery of QR.p. Scale bars in **b**, **d**, **e**, **g** & **h**, 5 µm. Statistical significance: ***P < 0.001 based on two-tailed Student’s *t-*test.

Daughter-cell growth potential appears to be associated with size asymmetry. The *C. elegans* Q.a neuroblast generated the large Q.ap cell that survives and differentiates into oxygen-sensing neurons; however, the small sibling Q.aa cell undergoes apoptosis^3^. Similarly, the asymmetric division of the *C. elegans* Q.p neuroblast generates a small posterior daughter-cell Q.pp that undergoes apoptosis and a large anterior daughter-cell Q.pa that divides and differentiates into neurons (Fig. 1a). Among 1090 somatically born *C. elegans* cells, 131 apoptotic cells result from ACDs. Intriguingly, the mammalian embryonic cerebral cortex development magnifies those in *C. elegans*: ~30% of newborn cells undergo programmed cell death^6^. It remains mysterious how these cells divide asymmetrically and determine the distinct daughter-cell sizes and fates.

This work shows that the *C. elegans* Q.p neuroblast employs a previously unrecognized strategy to accomplish its asymmetric cell division: Q.p asymmetrically positions its spindle and then continuously increases daughter cell asymmetry by Wnt gradient-guided unequal actin polymerization in the anterior cortex.

## Results

### Asymmetric cortical actin assembly in dividing Q.p neuroblasts

**Fig. 2:**
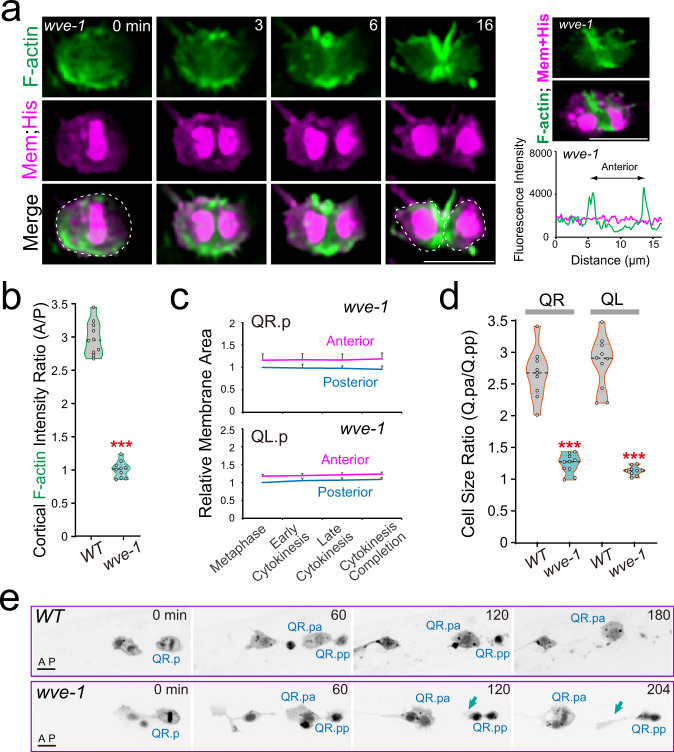
Asymmetric cortical actin polymerization regulates QR.p ACD. **a** Fluorescence time-lapse images of QR.p cytokinesis in the *wve-1* conditional knockout animals. Dotted lines show cell peripheries; and line-scan intensity plots of F-actin (green) with Myri-mCherry (magenta) signal around the periphery of another representative cell at the early cytokinesis. **b** Violin plots with all data points of the fluorescence intensity ratio of F-actin between the anterior and posterior of the cytokinetic QR.p in WT and *wve-1* conditional knockout worms (*n* = 10). **c** Quantification of the anterior and posterior plasma membrane area during QR.p (*n* = 10) and QL.p (*n* = 8) cytokinesis in *wve-1* conditional knockout animals. Data were normalized and compared to the metaphase posterior plasma membrane area. **d** Violin plots with all data points of daughter-cell size ratio of QR.p and QL.p in WT (*n* = 9–10) and *wve-1* (*n* = 9–10) conditional knockouts worms. **e** Fluorescence time-lapse images of the QR.pp fate in WT and *wve-1* conditional knockout animals. The QR.p lineage cell identities are denoted adjacent to the cells. Arrow: neurite outgrowth. Scale bars in **a** and **e**, 5 µm. Statistical significance: ***P < 0.001 based on two-tailed Student’s *t-*test.

At the start of Q.p neuroblast division, the mitotic spindle is posteriorly displaced, and non-muscle myosin II (NMY-2) homogeneously localizes around the ingressing furrow (Supplementary Videos S1–S2)^3^. Intriguingly, we observed that the anterior of the dividing cell progressively expanded during division, causing a continuous increase in the anterior-to-posterior cell size ratio from 1.2-fold at the onset of anaphase to 2.7-fold upon completion of cytokinesis (Figs. 1b, c, 2d; Supplementary Video S3). By measuring the two-dimensional area of the central cross-section of the dividing cells at metaphase and cytokinesis, we found that the plasma membrane was modestly enlarged 14% from metaphase to the completion of cytokinesis (Supplementary Fig. S1a), which suggests that membrane remodeling might be a significant driver of asymmetric membrane expansion in the anterior^7,8^. Importantly, these results cannot be easily explained by the displaced mitotic spindle and contractile ring but suggest that the increase in daughter cell asymmetry during cytokinesis may require an additional mechanism.

We sought to understand how a dividing cell increases daughter-cell size difference during cytokinesis. To follow actin dynamics during Q.p asymmetric division, we imaged the GFP-tagged actin-binding domain of moesin (GFP::moesinABD, Fig. 1d; Supplementary Fig. S1b, Videos S4–S5)^9^. Consistent with our previous results, actin filaments accumulated at the leading edge during QR.p cell migration on the right side of the animal (Supplementary Fig. S1c)^10^; when QR.p entered mitosis, this cell rounded up and lost the asymmetric distribution of F-actin in the cortex (Fig. 1d). Strikingly, at the end of metaphase, the fluorescence intensity of GFP::moesinABD in the anterior cortex was about 3.0-fold higher than that in the posterior cortex, revealing that the dividing QR.p cell asymmetrically assembled F-actin in the anterior cortex (Fig. 1d–f; Supplementary Video S4). Such a polarized F-actin distribution persisted throughout cytokinesis. Similarly, we observed the F-actin asymmetry during cytokinesis of the QL.p cell on the left side of the animal (Supplementary Fig. S1b, d, e, Video S5). Because QL.p does not migrate but asymmetrically assembles F-actin in the cortex when dividing (Supplementary Fig. S1c, d), the polarized actin polymerization is independent of cell migration.

To understand the molecular regulation of the cortical asymmetric actin assembly during Q.p division, we examined the dynamic distribution of the actin nucleation factor Arp2/3 complex (ARP2, *C. elegans* ARX-2::GFP), the actin nucleation-promoting WAVE complex (GFP:: WVE-1), and WASP protein (GFP::WSP-1). Using GFP knock-in animals, we found that the fluorescence intensity of ARX-2::GFP in the anterior was 2.7-fold higher than that of the posterior from metaphase to cytokinesis (Fig. 1f–h; Supplementary Fig. S2a–c, Video S6). WAVE and WASP accumulated 1.9- and 2.4-fold more at the anterior than at the posterior during cytokinesis (Fig. 1f, h; Supplementary Fig. S2a–d, Videos S7–S8), respectively. Thus, the anterior enrichment of Arp2/3 and its activating factors may lead to an asymmetric expansion of the plasma membrane in the anterior, resembling the extension of the leading edge in migrating cells.

### Arp2/3-mediated unequal cortical actin assembly regulates Q.p division

To determine the role of Arp2/3-nucleated actin polymerization during Q.p asymmetric division, we first examined Q.p division in animals defective in WAVE activity. The WAVE complex plays an essential role in *C. elegans* embryonic development. We used a somatic CRISPR-Cas9 platform^11^ to generate conditional mutants of *wve-1*, which encodes a WAVE subunit. As demonstrated in our previous Q cell migration study^10^, conditional mutation *wve-1-sg* effectively disrupted Arp2/3-nucleated actin polymerization in the leading edge. The *wve-1-sg* conditional mutation abolished asymmetric actin polymerization in the anterior cortex but did not perturb spindle positioning (Fig. 2a, b; Supplementary Fig. S3a–c, Video S9). Because actin filaments in the contractile ring are nucleated by formin but not Arp2/3^12^, actin filaments are assembled in the cleavage furrow (Fig. 2a; Supplementary Fig. S3a, Video S9), allowing Q.p cells to complete cytokinesis with a posteriorly shifted spindle. However, asymmetric membrane expansion was inhibited (Fig. 2c), decreasing the anterior and posterior daughter-cell size ratio from 2.7-fold to 1.2-fold (Fig. 2d). These results indicate that WAVE-Arp2/3-dependent actin polymerization in the anterior cortex increases daughter cell size asymmetry.

**Fig. 3:**
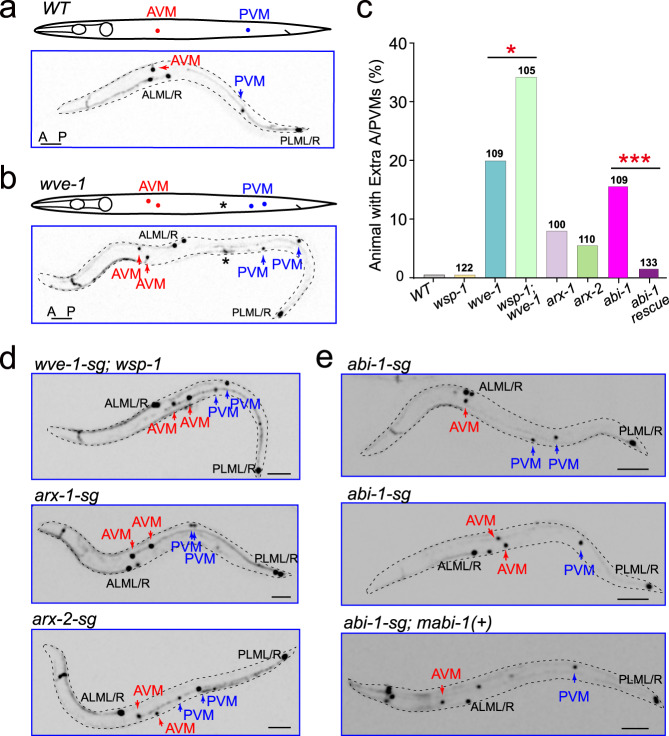
Defects in asymmetric actin assembly generated extra Q.p progenies. **a**, **b** Schematics and fluorescence inverted images of the AVM (red arrows) and PVM (blue arrows) neurons derived from Q.p lineages in WT (**a**) and *wve-1* conditional knockout (**b**) animals. A/PVM neurons were visualized using P*mec-4::gfp*. The cell identities are denoted adjacent to the cells; asterisks indicate the co-injected marker. Dotted black lines show the periphery of *C. elegans*. **c** Quantification of Q lineages with extra A/PVM neurons in WT (*n* > 300) and indicated mutant animals. *n* is as labeled. Statistical significance: *P < 0.05, ***P < 0.001 based on *χ*^2^ test. **d** Representative fluorescence inverted images of the A/PVM neurons in animals with indicated genotypes. **e** Representative fluorescence inverted images of the A/PVM neurons in *abi-1* conditional knockout or its Cas9-cleavage resistant transgene (*mabi-1(+)*) rescued animals. Scale bar in (**a**, **b**) and (**d**, **e**) 50 µm.

To address whether the change of daughter-cell size asymmetry perturbs cell fates, we performed time-lapse fluorescence recordings to follow daughter-cell behaviors after birth. In WT animals, the posterior daughters of Q.p (Q.pp) were engulfed and digested by neighboring cells within 150 min (Fig. 2e; Supplementary Fig. S3d, Video S10)^13^. In contrast, in a mutant for PAR-1-like kinase PIG-1, where Q.p divides equally^14^, Q.pa underwent regular division and differentiation into A/PVM and SDQL/R as in WT animals, whereas Q.pp survived and differentiated into either A/PVM or SDQL/R, thereby generating three neuron-like cells in late L1 larvae and an extra A/PVM or SDQL/R in adult animals^14^. Like *pig-1*, we showed that loss of WAVE did not perturb Q.pa development but caused Q.pp to escape apoptosis, and Q.pp differentiated into neuron-like cells that extended a long dendrite-like process (four out of six examined Q.pp cells) (Figs. 2e, 3b; Supplementary Fig. S3d, Video S11). Although our long-term live imaging results were informative, the methodology did not allow us to examine a large number of animals, and the data may not necessarily indicate the fate of the ectopically surviving cells in adult animals. Therefore, we examined GFP neuronal fate markers in the adult WAVE or Arp2/3 conditional knockout animals (more than 100 animals for each genotype). The WT animals only developed a single AVM and PVM mechanosensory neuron derived from the QR.p and QL.p cell lineage, respectively (Fig. 3a, c)^13^. In contrast, 20% of *wve-1-sg* or 16% of *abi-1-sg* (ABI-1, a subunit of the WAVE complex) conditional knockout animals produced extra AVM and PVM neurons (Fig. 3b, c, e). We cannot exclude the possibility that the ectopic neurons might result from off-target mutations by CRISPR-Cas9. Using *abi-1* as an example, we generated synonymous mutations in the CRISPR-Cas9 target site of *abi-1-sg*. The Cas9-cleavage resistant transgene fully rescued the extra neuron phenotype in *abi-1-sg* conditional mutant animals (Fig. 3c, e), suggesting that off-target effects are unlikely to account for these results.

*wsp-1(gm324)*, a null mutation for the alternative actin nucleation-promoting factor WASP in *C. elegans*, did not generate extra neurons or disrupt Q.p divisions (Fig. 3c). However, GFP::WSP-1 accumulated in the anterior cortex, same as WAVE-Arp2/3 (Fig. 1f, h; Supplementary Fig. S2, Video S8). We have previously shown that WASP partially compensated WAVE’s loss during *C. elegans* Q cell migration^10^, prompting us to determine whether WAVE and WASP have redundant functions in ACDs. By generating a *wve-1-sg, wsp-1(gm324)* double-mutant strain, we found that the penetrance of the extra neuron phenotype was significantly enhanced to 34% (Fig. 3c, d), revealing a parallel role of WASP and WAVE in this process. *wve-1-sg* conditional mutations reduced QR.p cell migration but did not affect QL.p cell location, as QL.p is a nonmigratory cell in WT animals^10,13^. We also found that *wve-1-sg* conditional mutations abolished asymmetric actin polymerization in the anterior cortex of QL.p, but did not perturb spindle positioning in QR.p or QL.p (Supplementary Fig. S3a–c), suggesting that cell migration and ACDs might be independent. Both the *arx-1* and *arx-2* conditional knockout mutants displayed the extra neuron phenotype but were more subtle than *wve-1-sg* single- or *wve-1-sg; wsp-1(gm324)* double-mutant animals (Fig. 3c, d). We found that *arx-2-sg* conditional knockout efficiently depleted ARX-2::GFP (Supplementary Fig. S3e). The *arx-2-sg* animals reduced Q cell migration to a less severe level than *wve-1-sg* animals^10^, suggesting that residual Arp2/3 complex activity might be responsible for the partial function. Together, our results show that disruption of polarized cortical actin polymerization increases posterior daughter-cell size, allowing Q.pp to survive and differentiate into neuron-like cells.

### The equal actin polymerization in the cortex causes symmetric cell divisions

**Fig. 4:**
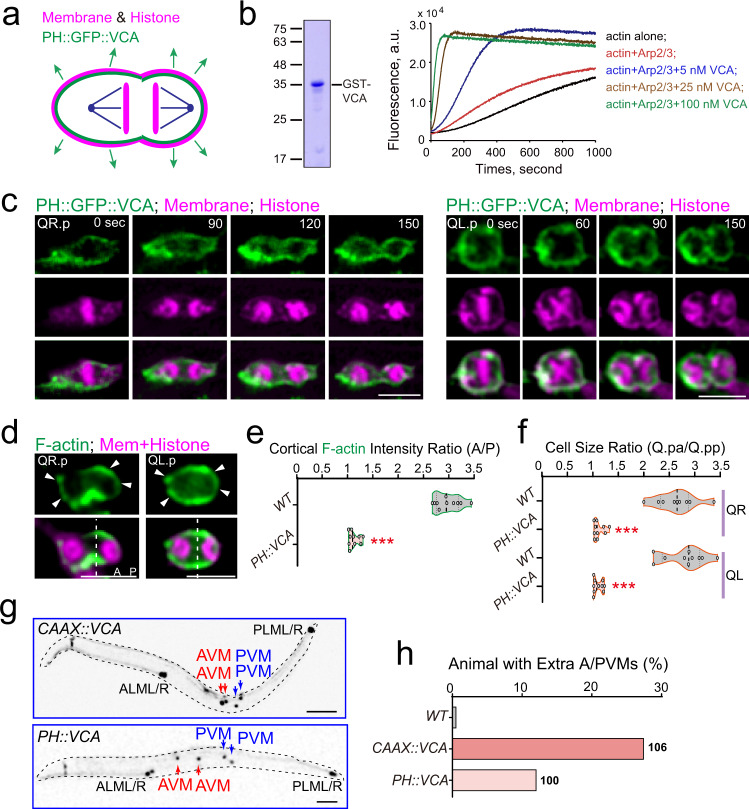
Symmetric cortical actin polymerization disrupts Q.p ACD. **a** Schematic of VCA domain overexpression in Q.p. The PH domain targeted the VCA domain in the cortex symmetrically. **b** Spectrofluorimetry assay using 10% pyrene-labeled actin to monitor actin polymerization. Protein concentrations are indicated. **c** Fluorescence time-lapse images of PH::GFP::VCA dynamics during Q.p cytokinesis. **d** Representative images of GFP-tagged moesinABD (F-actin) and mCherry-tagged plasma membrane (Mem) and histone (His) of cytokinetic Q.p in *PH::VCA* transgenic worms. The dotted white line indicates contractile ring position, and the white arrowhead indicates F-actin enrichment at the cell pole cortex. **e** Violin plots with all data points of the fluorescence intensity ratio of F-actin between the anterior and posterior of the cytokinetic QR.p in WT and *PH::VCA* transgenic worms (*n* = 10). Statistical significance: ***P < 0.001 based on two-tailed Student’s *t*-test. **f** Violin plots with all data points of daughter-cell size ratio of QR.p and QL.p in WT (*n* = 9–10) and *PH::VCA* transgenic (*n* = 10) worms. Statistical significance: ***P < 0.001 based on two-tailed Student’s *t*-test. **g** Representative fluorescence inverted images of the A/PVM neurons in animals with indicated transgenic background. **h** Quantification of Q lineages with extra A/PVM neurons in WT (*n* > 300) and indicated mutant animals. *n* is as labeled. Scale bar in (**c**) and (**d**), 5 µm; in (**g**), 50 µm.

We reasoned that if polarized actin polymerization in the anterior cortex is vital, ectopic actin assembly in the posterior cortex may change the asymmetry of daughter-cell size and fate. To test this idea, we sought to artificially target the VCA domain of the *C. elegans* WAVE that can constitutively activate Arp2/3 throughout the entire plasma membrane using a membrane-targeting signal (the PH domain of GRP-1 or the CAAX motif of the Rac GTPase MIG-2) (Fig. 4a). We first prepared the recombinant GST-tagged worm VCA domain and showed that this domain stimulated Arp2/3-based actin polymerization in a dose-dependent manner using a pyrene-actin polymerization assay in vitro (Fig. 4b)^15,16^. Indeed, the equal distribution of PH::GFP::VCA in the dividing Q.p cell membrane promoted actin assembly in the anterior and posterior cortex, causing a more symmetric division (Fig. 4c–f; Supplementary Fig. S3b, Video S12). The expression of PH-tagged or CAAX-tagged VCA during the Q.p division generated extra AVM and PVM neurons in 12% or 27% transgenic animals, respectively (Fig. 4g, h). These results provide further evidence that the asymmetry of cortical actin polymerization during cytokinesis contributes to the generation of daughter cells of distinct sizes and fates.

### Wnt signaling directs asymmetric actin assembly during Q.p division

We aimed to identify the environmental cue that directs asymmetric cortical actin assembly during ACDs. Wnt signaling plays an evolutionarily conserved role in establishing anterior–posterior polarity^17,18^. During Q cell development, loss of Wnt components, including Wnt ligands, the Frizzled receptor, and downstream transcriptional factor MAB-5, redirects QL progenies’ posterior migration towards anterior^19,20^. Early research from the Kenyon lab found that heat shock of the *mab-5* gene expression reversed the migration directionality of cells in the QR lineage^21,22^. Still, no defects in Q cell asymmetric division are known in *mab-5* loss- or gain-of-function mutant animals^21^, suggesting that MAB-5 acts in nuclei to regulate a gene transcription program underlying cell migration but may not direct ACDs from the cell cortex.

**Fig. 5:**
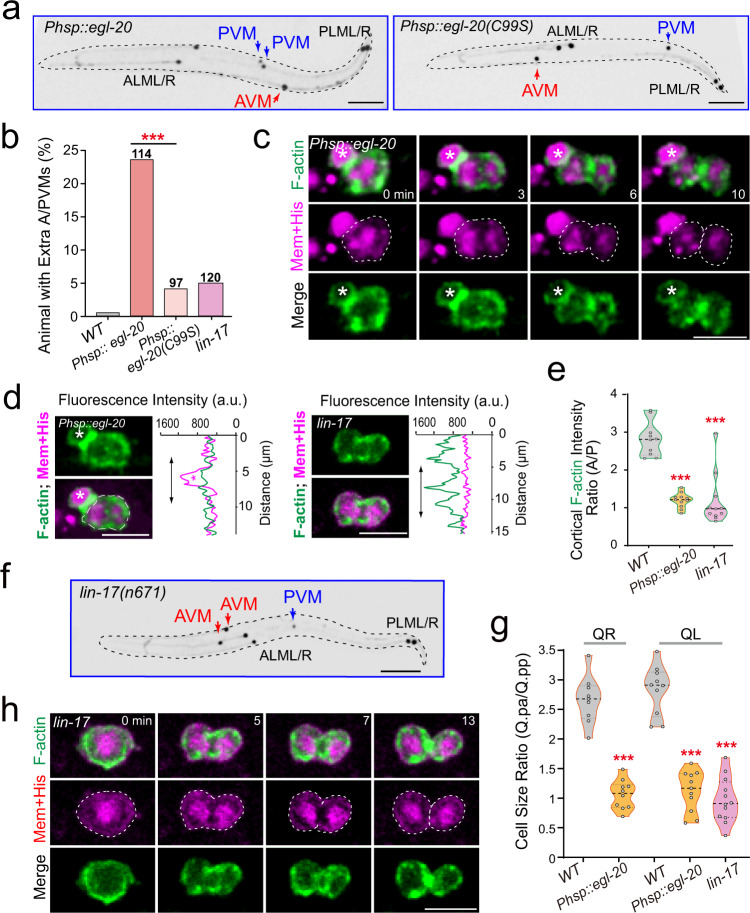
Wnt signaling regulates Q.p ACD. **a** Fluorescence inverted images of the A/PVM neurons in animals with the indicated P*hsp-16.2::egl-20* transgene. **b** Quantification of Q lineages with extra A/PVM neurons in WT (*n* > 300) and indicated mutant animals. *n* is as labeled. Statistical significance: ***P < 0.001 based on *χ*^2^ test. **c** Fluorescence time-lapse images of QL.p cytokinesis in the P*hsp-16.2::egl-20* transgenic animals. **d** Image and line-scan intensity plots of F-actin (green) with Myri-mCherry (magenta) signal around the same cell’s periphery at the early cytokinesis of QL.p cells in P*hsp-16.2::egl-20* transgenic or *lin-17(n671)* mutant animals. Asterisks indicate neighboring Q cells. **e** Violin plots with all data points of the fluorescence intensity ratio of F-actin between the anterior and posterior of the cytokinetic QL.p in WT, P*hsp-16.2::egl-20* transgenic or *lin-17(n671)* mutant worms (*n* = 10–11). **f** Fluoresce*n*ce inverted images of the A/PVM neurons in *lin-17(n671)* mutants. **g** Violin plots with all data points of daughter-cell size ratio of QR.p and QL.p in WT, P*hsp-16.2::egl-20* transgenic or *lin-17(n671)* mutant worms (*n* = 9–12). **h** Fluoresce*n*ce time-lapse images of QL.p cytokinesis in the *lin-17(n671)* mutants. Statistical significance in (**b**, **e**, **g**): ***P < 0.001 based on two-tailed Student’s *t*-test. Scale bar in (**c**, **d**, **h**), 5 µm; in (**a**, **f**), 50 µm.

We wondered whether Q.p ACD used Wnt components other than MAB-5 to establish polarity. The *C. elegans* genome encodes five Wnt ligand genes, and single mutations do not affect Q cell asymmetric divisions. Functional redundancy is an obstacle to dissecting the role of Wnt in *C. elegans* neural development^20^, and no extra touch neurons were produced in either *egl-20*^21^ or compound Wnt mutants^23^. On the other hand, it is the elaborate Wnt gradient that activates the downstream signaling cascade. Inspired by early studies in which a global Wnt/EGL-20 overexpression disrupted Q cell migration directionality^24^, we switched to the same heat-shock inducible Wnt overexpression strategy to disrupt the Wnt gradient. The QR progenies underwent the anterior migration in the EGL-20 transgenic animals without heat-shock treatment; however, they migrated towards the tail of the animal after heat shock (Fig. 5a, 64.9%, *n* = 114), which indicates that global expression of a Wnt ligand effectively perturbs anterior–posterior polarity in *C. elegans* larvae.

Next, we followed Q.p cell asymmetric division in EGL-20 transgenic animals. The dividing Q.p cells assembled actin filaments in the anterior and posterior cortex and eventually generated two daughter cells with similar sizes (Fig. 5c–e, g; Supplementary Fig. S4a, b, Video S13). Consistently, 23.7% of these animals developed extra A/PVM neurons (Fig. 5a, b). To exclude Wnt overexpression artifacts or side effects of heat shock, we used the heat-shock system to overexpress an EGL-20 (C99S) loss-of-function mutation^21^. After heat shock, the EGL-20 (C99S) transgenic animals generated significantly fewer extra A/PVM neurons than WT EGL-20 (Fig. 5a, b), providing a negative control for the EGL-20 overexpression results. These data show that Wnt gradient disruption causes Q.p cells to divide symmetrically, allowing some of their apoptotic small daughter cells to survive and differentiate.

We next examined whether a Wnt receptor perceived the Wnt signal during Q.p cell asymmetric division. The Frizzled receptor LIN-17 was previously shown to transduce the Wnt signal to QL descendants to ensure their posterior migration^19,25^, making it a candidate receptor for asymmetric Q.p division. Like WAVE conditional mutants, the loss-of-function mutation of LIN-17 in *lin-17(n671)* mutant animals caused an even distribution of the actin cytoskeleton in the cortex of dividing Q.p cells, generating two daughter cells with similar sizes (Fig. 5b, d, e, g, h). Q.p progenies in WT animals did not develop any extra neurons derived from Q.p cell lineages (*n* > 100); however, adult *lin-17(n671)* mutant animals formed ectopic A/PVM neurons (Fig. 5b, f, 3% for AVM; 6% for PVM, *n* = 120), indicating an involvement of the Frizzled receptor LIN-17 in ACD. Loss of WAVE or Arp2/3 generates extra neuron phenotype at modest penetrance. Likewise, we noticed that the extra neuron penetrance is much lower than ACD in *lin-17* mutant animals. This discrepancy is unlikely to result from the functional redundancy of Frizzled receptors. Instead, loss of size asymmetry may not be sufficient to affect apoptosis or cell fate. An ectopic gain of a neuron from an apoptotic cell probably requires both symmetric division and apoptosis inhibition. Supporting this idea, neither *pig-1* disrupting ACD nor *ced-3* inhibiting caspase-3 generates high penetrance of extra neurons; however, their double-mutant animals displayed extra neuron defects with over 80% penetrance^14^.

**Fig. 6:**
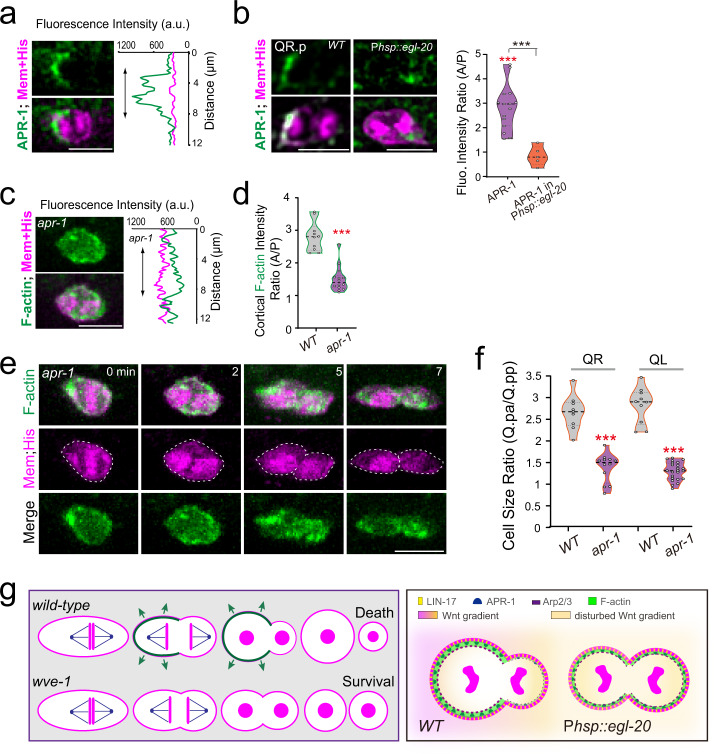
Wnt signaling directs asymmetric actin assembly during Q.p ACD. **a** Representative images of GFP-tagged APR-1, with the mCherry-tagged plasma membrane and histone in cytokinetic QL.p, and the corresponding line-scan intensity plots (green) with Myri-mCherry (magenta) signal around the periphery of QL.p. **b** Representative images of GFP-tagged APR-1, and violin plots with all data points of the fluorescence intensity ratio of APR-1 between the anterior and posterior of the cytokinetic QL.p in WT (*n* = 15) and P*hsp-16.2::egl-20* transgenic (*n* = 5) worms. **c** Representative image and line-scan intensity plots of F-actin (green) with Myri-mCherry (magenta) signal around the same cell’s periphery at the early cytokinesis of QL.p cells in *apr-1(ok2970)* mutant worms. **d** Violin plots with all data points of the fluorescence intensity ratio of F-actin between the anterior and posterior of the cytokinetic QL.p in WT (*n* = 10) or *apr-1(ok2970)* mutant (*n* = 14) worms. **e** Fluorescence time-lapse images of QL.p cytokinesis in the *apr-1(ok2970)* mutant animals. **f** Violin plots with all data points of daughter-cell size ratio of QR.p and QL.p in WT (*n* = 9–10) and *apr-1(ok2970)* mutant (*n* = 12–23) worms. **g** Proposed models. Left: Polarized actin polymerization in the cortex may generate force to promote asymmetric membrane expansion during Q.p cytokinesis, producing two unequal daughter cells. Disruption of polarized actin polymerization (e.g., in the *wve-1* mutant) led to more symmetric cell division. Right: the Wnt signal polarizes the actin polymerization through LIN-17 and APR-1. Statistical significance in (**b**, **d**, **f**): ***P < 0.001 based on two-tailed Student’s *t*-test. Scale bar in (**a**, **c**, **e**), 5 µm.

To understand how the Frizzled receptor regulates asymmetric cortical actin assembly, we examined LIN-17 dynamic distribution during Q.p cell division. Using a functional GFP translational reporter, we found that LIN-17::GFP fluorescence evenly distributed throughout the plasma membrane of the dividing Q.p cell (Supplementary Fig. S4c, d), raising the question of how to activate the intracellular downstream components asymmetrically. A recent study systematically tagged *C. elegans* Wnt signaling components with a fluorescent protein at their endogenous loci^26^. We genetically crossed Q cell markers into these knock-in animals and then performed live-cell imaging to examine which component(s) were asymmetrically distributed in the dividing Q.p cells. We found that the tumor suppressor Adenomatous Polyposis Coli (APC) protein homolog^27^, APR-1 in *C. elegans*, was 2.5-fold enriched at the anterior cortex compared to the posterior during Q.p division (Fig. 6a, b; Supplementary Figs. S4e, f, S5a). In support of the notion that APC functions downstream of the Wnt signaling perception, the enrichment of APC on the cortex of dividing Q.p was reduced from 2.5-fold to 0.8-fold in the presence of ectopically expressed EGL-20 (Fig. 6b; Supplementary Figs. S4f,S5b). Because the disruption of Wnt gradient in P*hsp::egl-20* worms impaired Q cell migration and caused QR.a and QR.p cells to have physical contact, the quantification of APR-1 fluorescence intensity cannot be accurate if the signals from both cells overlap. Nevertheless, we quantified the ARP-1 signal from movies in which we can clearly distinguish the fluorescence from each cell. Despite the low number, the quantifications consistent with other lines of evidence, supporting that APR-1 functions downstream of Wnt signaling in regulating actin asymmetry during Q.p cell ACD. Consistently, the Wnt signal directs the polarized distribution of APC during oriented cell division in the *C. elegans* four-cell-stage embryo, even though Wnt-APC is involved instead in spindle orientation in such context^26^.

We examined actin distribution in the dividing Q.p cells in the APC mutant *apr-1 (ok2970)* larvae. Asymmetric localization of F-actin and cell size in WT cells became symmetrical without APC (Fig. 6c–f; Supplementary Fig. S4g, h). Our long-term fluorescence imaging showed that the Q.pa cell developed normally, but all Q.pp cells escaped from apoptosis and formed neuron-like cells in the late L1 larvae (Supplementary Fig. S5c, *n* = 8). In vitro biochemical studies have shown that APC directly promotes Arp2/3-dependent actin polymerization^28^, linking *C. elegans* APC/APR-1 asymmetry to polarized actin assembly in the anterior. A proteomic analysis described that the *C. elegans* APC/APR-1 protein directly binds to WAVE subunit ABI-1^29^, raising a non-exclusive possibility that APC may recruit the WAVE complex to the anterior of the dividing Q.p cells. Consistently, a conditional *abi-1-sg* mutant for the APC-binding ABI-1 subunit of the WAVE complex produced ectopic A/PVM neurons (Fig. 3c, e). Hence, in response to the Wnt signal, APC asymmetrically distributes to the anterior cortex to promote polarized actin assembly during ACDs.

## Discussion

This work shows that asymmetric cortical expansion, produced by unequal actin polymerization in the cortex, increases the asymmetry of daughter cells in *C. elegans* neuroblasts. We propose that an outward-expanding anterior pole may allow the posterior cytoplasm to move through the furrow, increasing anterior cell size and reducing rear cell size (Fig. 6g). Our results show that branched actin networks are asymmetrically assembled in the cell cortex and that their nucleation factor Arp2/3 displays a consistent polarized distribution throughout the cortex (Fig. 1d–h; Supplementary Figs. S1b, d, e, S2). Given that formins and formin-nucleated linear actin filaments usually function in the contractile ring rather than at the cell cortex^12^, we suspect that the Arp2/3-based branched actin network is the primary player in asymmetric membrane expansion. Future studies will determine whether formins and linear actin filaments regulate cortical dynamics during ACDs. The Insall lab reported that, during *Dictyostelium* cytokinesis, the activation of the actin nucleation factor SCAR/WAVE during mitosis is essential for driving myosin-independent cytokinesis^30^. Although Q.p cytokinesis requires myosin II in the contractile ring, these studies indicate that parallel molecular pathways drive cytokinesis in different types of animal cells.

Together with earlier studies^3,14^, our results suggest an intriguing connection between cell size and apoptotic cell fate. According to our data, small cells undergo apoptosis, whereas middle- or larger-sized cells survive and differentiate into neurons. Perhaps, a cell requires a minimum volume and content to live. However, in mutant animals defective in the Caspase-3 CED-3 or the cell death-inducing factor EGL-1, ectopic neurons are derived from apoptotic Q.pp cells, whose sizes are identical to those in WT animals^14^, indicating that inhibition of apoptosis promotes cell survival and differentiation. An increase of Q.pp cell sizes in the *pig-1* mutant generated extra A/PVM neurons with less than 40% penetrance, and loss of CED-3 caused extra A/PVM neurons at ~20% penetrance. Strikingly, the double mutations of *pig-1* and *ced-3* led to the additional A/PVM phenotype with more than 80% penetrance, indicating the increase of cell size and inhibition of programmed cell death function in parallel to promote cell survival and differentiation.

This study expands the functional repertoire of Wnt components in ACDs. Wnt ligands have been well known for their critical roles of ACDs in the *C. elegans* early embryos, and previous studies also uncovered the function of Wnt pathway components in the *C. elegans* epithelial ACDs. Our work identified several Wnt elements, such as the Frizzled receptor and downstream APC protein, as essential regulators for neuroblast ACD. During all these ACDs, Wnt components most likely function with cortical proteins from the cell cortex but not through transcriptional regulations. We noticed that the neighboring epithelial cells (i.e., the hyp7 cell or seam cells) surrounding Q cells have high fluorescence signals from the APR-1::GFP knock-in reporter. While this work described the asymmetric localization and function of APR-1 in dividing Q.p cells, the polarized distribution of APR-1 was observed in seam cells and embryonic cells^31,32^. We suggest that an asymmetric distribution and function of APR-1 might also occur in Q.a cells, likely generating a cortical expansion domain along with the NMY-2-based contractile region^3^ to produce two different-sized daughter cells.

Polarized actin polymerization that expands daughter-cell size in Q.p complements myosin contractility that shrinks daughter-cell size in Q.a, indicating that asymmetric cell division may be more complex than currently appreciated. Curiously, in spiralians, the formation of a polar lobe sets up asymmetry in the first two cleavages, and Arp2/3 is required for defining the cortical zone that will be sequestered into the polar lobe, demonstrating a general role of Arp2/3 in polarity establishment^33^. Moreover, our results show that spindle displacement and polarized actin polymerization function in concert to promote asymmetric cell division. For the Q.p division, spindle displacement contributes to 1.2-fold cell size asymmetry at metaphase, whereas polarized actin polymerization further increases the asymmetry to 2.7-fold during cytokinesis. The disruption of polarized actin polymerization by WAVE mutations did not perturb spindle displacement (Supplementary Fig. S3c), and produced 1.2-fold cell size asymmetry in Q.p (Fig. 2d). Hence, a single cell uses two sequential physical mechanisms, namely spindle displacement and subsequent polarized cortical expansion, to generate asymmetric cell division. Considering that asymmetric cortical actin polymerization occurs before spindle displacement (Supplementary Video S4), these two processes appear to be independent, and spindle displacement could act in parallel to asymmetric cortical actin polymerization.

Future studies will understand the molecular and cellular regulation of neural stem cell asymmetric divisions in brains. Although 30% of the mammalian newborn neural progenitor cells undergo programmed cell death^6^, how ACDs generate them remains mysterious. Neither do we understand how ACDs determine their apoptotic cell fate. Findings from simple model organisms such as *C. elegans* and *Drosophila* can provide a conceptual framework for addressing similar questions during brain development.

## Materials and methods

### *C. elegans* strains, DNA manipulations, and transgenesis

*C. elegans* strains were maintained on NGM plates seeded with the *Escherichia coli* strain OP50 at 20 °C. The Bristol variety N2 is the wild-type strain. *C. elegans* strains, primers, and plasmids used in this study are listed in Supplementary Table S1.

We constructed transgenes using the In-Fusion cloning protocol (Clontech). The In-Fusion cloning strategy is based on the 15 nt homologous sequences at the ends of the vector and linearized fragments. We used the P*mec-4::gfp* reporter to visualize the AVM/PVM neurons in young adult animals and used the *egl-17* promoter to drive the target gene for Q cell-specific expression. The cDNA that encodes the WAVE (*wve-1*)’s VCA domain was amplified from the *C. elegans* cDNA library of the mixed developmental stage.

We performed microinjection to express transgenes in *C. elegans*. The transgene plasmids, together with an injection marker plasmid (P*odr-1::dsRed* or *unc-76(+)*) were injected into the syncytial gonad of N2 or *unc-76* mutants. To avoid potential artifacts caused by the overexpression of transgene, we injected the transgene plasmid at the lowest possible concentration. We injected 10–20 ng/μL DNA constructs into 30–50 young adult animals and chose three transgenic lines whose transmission rates of extrachromosomal arrays are > 60%.

### Live-cell imaging

To maximize the efficiency of live-cell imaging, we synchronized *C. elegans* by collecting ~100 young adult worms on the NGM plate to lay eggs for 2 h and then removing the adults. The eggs were maintained at 20 °C for ~16 h to develop into L1 larvae.

For live-cell imaging, L1 worms were anesthetized with 0.1 mmol/L levamisole in M9 buffer and mounted on 3% agarose pads at 20 °C. Live-cell imaging was performed using an Axio Observer Z1 microscope (Carl Zeiss MicroImaging, Inc.) equipped with a 100×, 1.45 N.A. objective, an EM CCD camera (Andor iXon+ DU-897D-C00-#BV-500), and the 488-nm and 568-nm lines of a Sapphire CW CDRH USB Laser System attached to a spinning disk confocal scan head (Yokogawa CSU-X1 Spinning Disk Unit). Time-lapse images were acquired with an exposure time of 200 ms every 30 s using the μManager (https://www.micro-manager.org/) and processed with ImageJ software.

### Quantifications and statistical analysis

ImageJ software was used to circumscribe the fluorescence field and measure the fluorescence intensity. For all the intensity measurements, the background was subtracted. For F-actin, ARX-2, WVE-1, WSP-1, and APR-1 distribution analysis during Q.p cytokinesis, we quantified the fluorescence intensity ratio of GFP on the anterior and posterior cortex; the mCherry fluorescence intensity ratio of the plasma membrane was used as the control. When we quantified the membrane area of anterior and posterior parts of dividing the Q.p cell, the equatorial plate or contractile furrow was used as a boundary. We used the Student’s *t*-test and *χ*^2^ analysis to determine the significant differences indicated in the figure legends.

### VCA (WVE-1) and actin preparation

Plasmids were transformed into BL21 of *E. coli* (DE3). Recombinant GST-tagged VCA proteins were expressed by induction with 0.4 mM IPTG at 16 °C overnight. Bacterial cells were collected and disrupted using an Ultrasonic Cell Disruptor. GST-VCA was purified by glutathione-affinity chromatography using a standard purification protocol and further purified by ion exchange and gel filtration chromatography (Source-15Q/15 S and Superdex21 200). Purified proteins were dialyzed against 50 mM Tris-HCl, pH 8.0, frozen in liquid nitrogen, and stored at –80 °C. The bovine Arp2/3 complex was purchased from Cytoskeleton, Inc. Actin was prepared from acetone powder of rabbit skeletal muscle, purified by Sephacryl S-300 chromatography, and labeled on Cys-374 with pyrene iodoacetamide fluorometry assay to monitor the kinetic actin-polymerization process as previously described^34,35^.

### Actin-polymerization assays

We performed the actin-polymerization assays using previously described methods^36,37^. We mixed 4 μM rabbit muscle actin (10% pyrene-labeled) with or without different concentrations of VCA domain in polymerizing buffer (10 mM imidazole-HCl pH 7.0, 10.2 mM CaCl_2_, 0.2 mM ATP, 0.2 mM DTT, 50 mM KCl, 1 mM MgCl_2_, 1 mM EGTA, and 0.02% NaN3), and the total volume was up to 150 μL. The fluorescence intensity was instantaneously monitored using a QuantaMaster Luminescence QM 3 PH fluorimeter (Photon Technology International) at room temperature for 1000 s with 365 and 407 nm excitation and emission wavelengths, respectively.

## Supplementary information

Supplementary Figures and Tables

Supplementary Video 1

Supplementary Video 2

Supplementary Video 3

Supplementary Video 4

Supplementary Video 5

Supplementary Video 6

Supplementary Video 7

Supplementary Video 8

Supplementary Video 9

Supplementary Video 10

Supplementary Video 11

Supplementary Video 12

Supplementary Video 13

## Data Availability

Source data are provided with this paper. All other datasets generated and analyzed in the current study are available from the corresponding author upon reasonable request.
