# Supplementary material for: Wnt signaling polarizes cortical actin polymerization to increase daughter cell asymmetry
Source: Cell Discov. 2022 Mar 1;8:22. doi: 10.1038/s41421-022-00376-4 (PMC8885824; doi:10.1038/s41421-022-00376-4)
Supplement: Supplementary file 1 — Supplementary Figures and Tables [file 41421_2022_376_MOESM1_ESM.pdf]

Supplemental Fig. S1

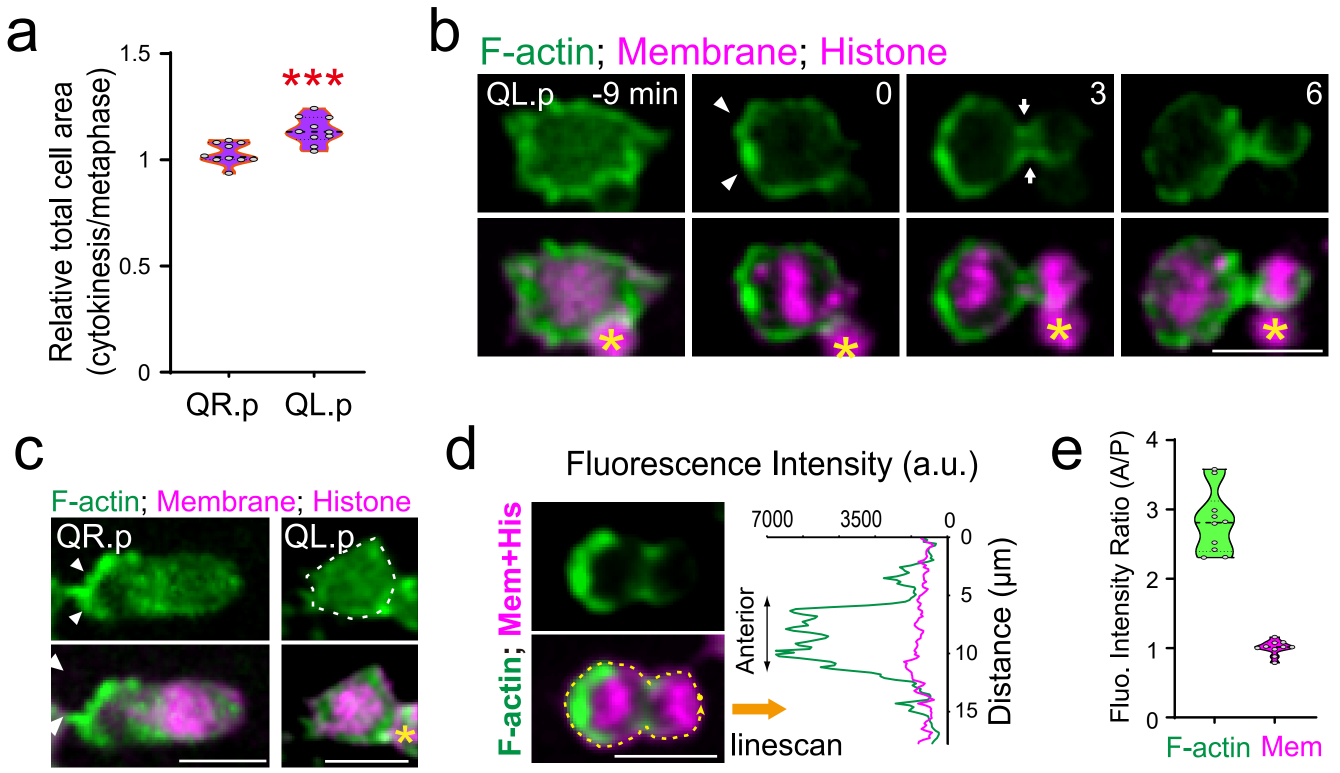

Supplemental Figures

Supplementary Fig. S1. Asymmetric cortical actin distribution during QL.p cytokinesis.

(a) The relative total cell area changes after cytokinesis. Statistical significance: ***p < 0.001 based on two-tailed Student's t-test (N = 10). **(b)** Fluorescence time-lapse images of GFP-tagged moesinABD (F-actin) and mCherry-tagged plasma membrane and histone during QL.p cytokinesis. Arrowheads indicate cortical fluorescence enrichment; arrows in (b) and (c) indicate fluorescence enrichment on the contractile ring; asterisks in (b) and (c) indicate neighboring Q cells. **(c)** Representative images of GFP-tagged moesinABD (F-actin) and mCherry-tagged plasma membrane and histone in interphase QR.p and QL.p. Dotted lines show cell peripheries. **(d)** Line-scan intensity plots (a.u.) of the F-actin (green) and Myri-mCherry (magenta) signal around the periphery of QL.p in the inserted images. The trace begins from the posterior of QL.p and moves counterclockwise along the cell periphery to the anterior and then back to the posterior. **(e)** Violin plots with all data points of the fluorescence intensity ratio of F-actin (green) and plasma membrane (magenta) between the anterior and posterior of the cytokinetic QL.p (N = 10). Statistical significance in (a) and (e): ***p < 0.001 based on two-tailed Student's t-test. Scale bar in (b)-(d), 5 µm.

Supplemental Fig. S2

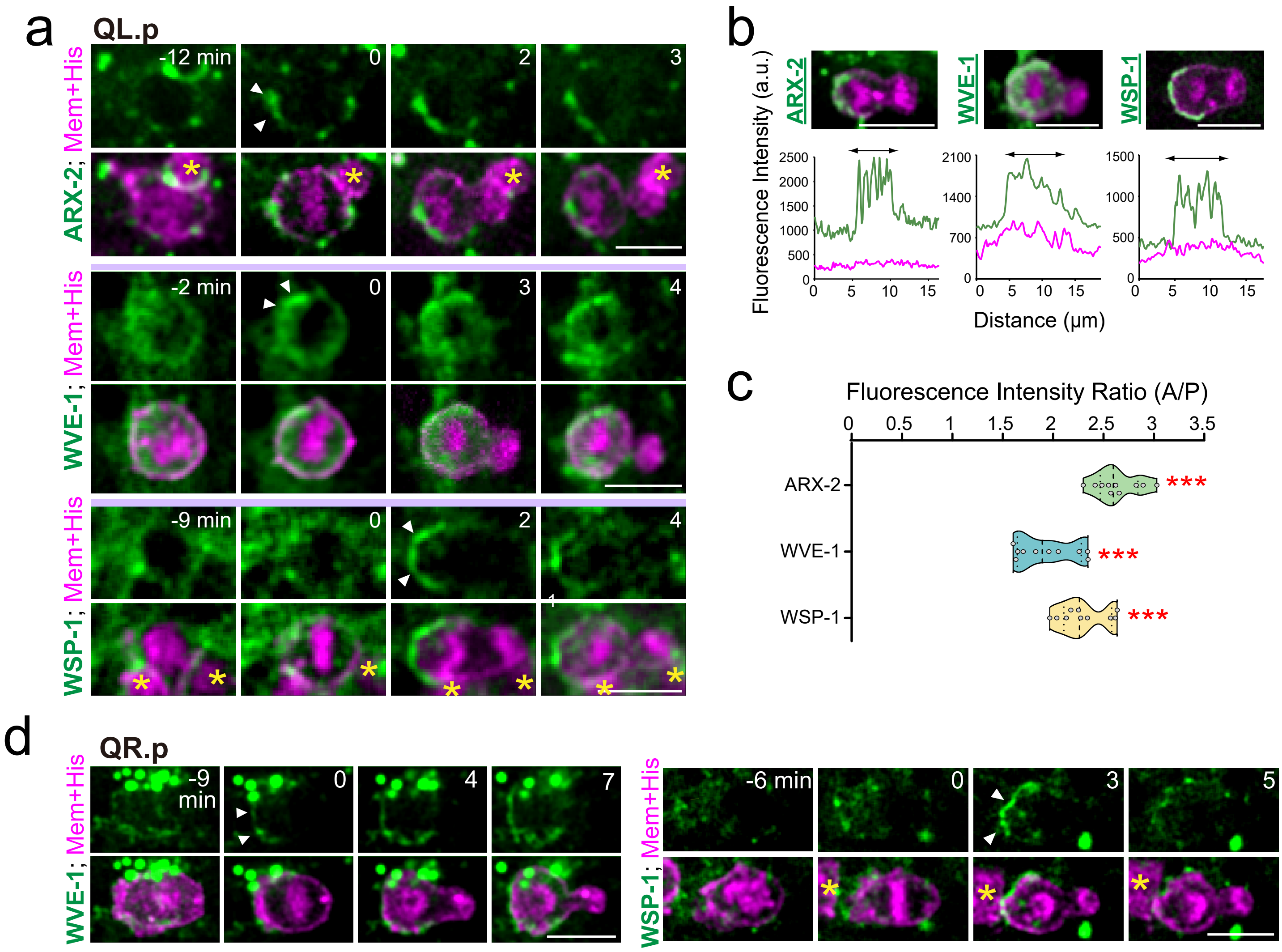

Supplementary Fig. S2. Asymmetric cortical actin polymerization during QL.p cell division.

(a) Fluorescence time-lapse images of GFP-tagged ARX-2, WVE-1, WSP-1, and mCherry-tagged plasma membrane and histone during QL.p cytokinesis. Arrowheads indicate cortical fluorescence enrichment; arrows indicate fluorescence enrichment on the contractile ring; asterisks indicate neighboring Q cells. **(b)** Representative image and line-scan intensity plots (a.u.) of the F-actin, ARX-2, WVE-1, WSP-1 (green), and Myri-mCherry (magenta) signal around the periphery of QL.p in the inserted images. The trace begins from the posterior of QL.p and moves counterclockwise along the cell periphery to the anterior and then back to the posterior. **(c)** Violin plots with all data points of the fluorescence intensity ratio of ARX-2, WVE-1, and WSP-1 (as indicated) between the anterior and posterior of the cytokinetic QL.p (N = 10). Statistical significance: ***$p < 0.001$ based on two-tailed Student's t-test. **(d)** Fluorescence time-lapse images of GFP-tagged WVE-1, WSP-1, and mCherry-tagged plasma membrane and histone during QR.p cytokinesis. Arrowheads indicate cortical fluorescence enrichment; arrows indicate fluorescence enrichment on the contractile ring; asterisks indicate neighboring Q cells. Scale bars in (a), (b) and (d), 5 µm.

Supplemental Fig. S3

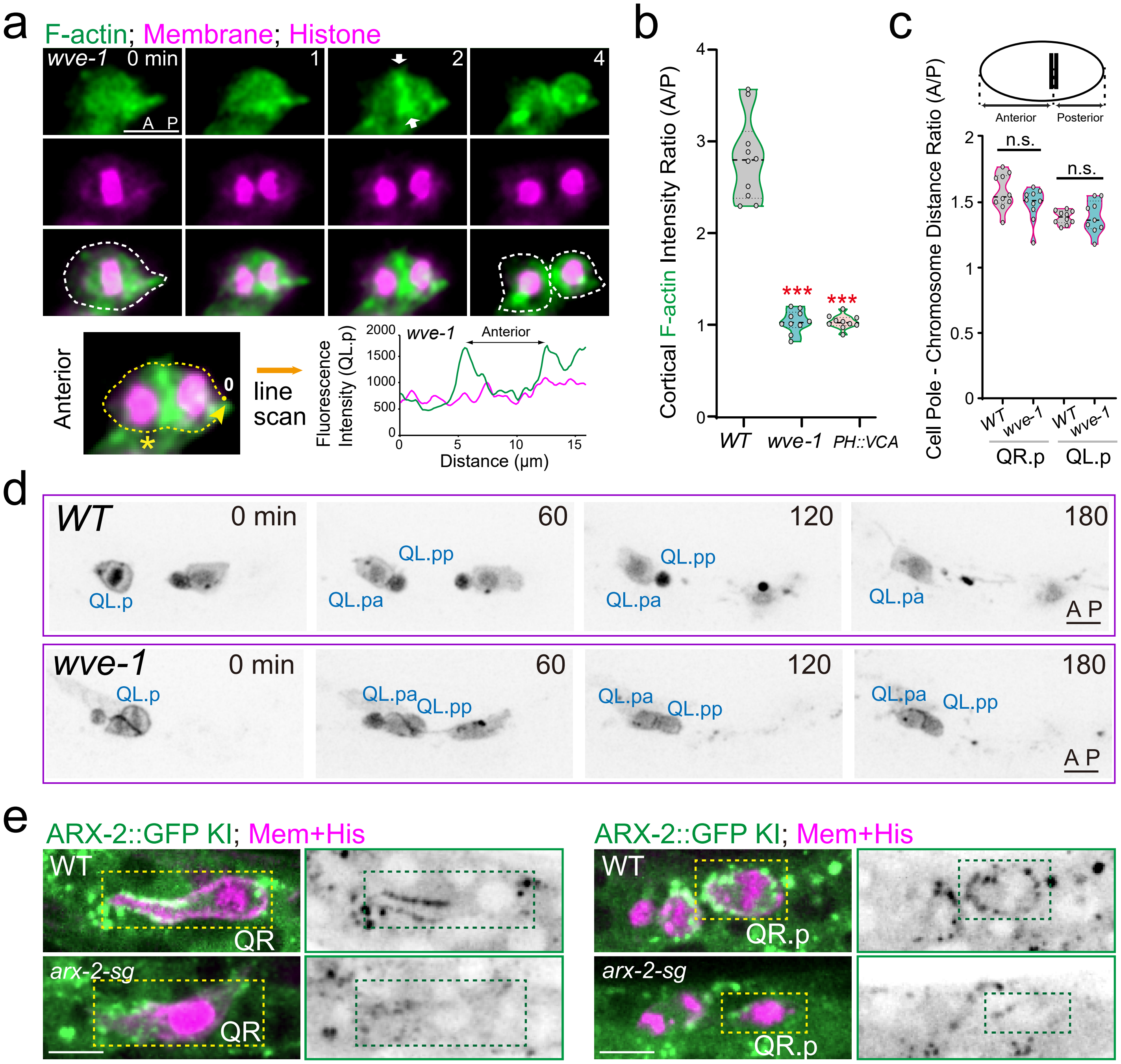

Supplementary Fig. S3. Asymmetric cortical actin polymerization regulates Q.p ACD.

(a) Fluorescence time-lapse images of QL.p cytokinesis in the *wve-1* conditional knockout (dotted lines show cell peripheries); and line scan intensity plots (right) of GFP-tagged moesinABD (F-actin) and mCherry-tagged plasma membrane and histone of cytokinetic QL.p in *wve-1* conditional knockout animals. **(b)** Violin plots with all data points of the fluorescence intensity ratio of F-actin between the anterior and posterior of the cytokinetic QL.p in WT, *wve-1* conditional knockouts, and *PH::VCA* transgenic worms (N = 10). Statistical significance: ***$p < 0.001$ based on two-tailed Student's t-test. **(c)** Quantification of cell pole to chromosome distance at metaphase in WT (N = 10) and *wve-1* (N = 9-10) conditional knockouts. n.s. no significance, based on two-tailed Student's t-test. **(d)** Fluorescence time-lapse images of the QL.pp fate in WT and *wve-1* conditional knockout animals. The QL.p lineage cell identities are denoted adjacent to the cells. **(e)** Representative images of GFP-tagged ARX-2 and mCherry-tagged plasma membrane and histone in QR (left) or QR.p (right) in WT or *arx-2* conditional knockout animals. Cells of interest are boxed with dotted lines, and cell identities are denoted adjacent to the cells. Scale bars in (a), (d) & (e), 5 μm.

Supplemental Fig. S4

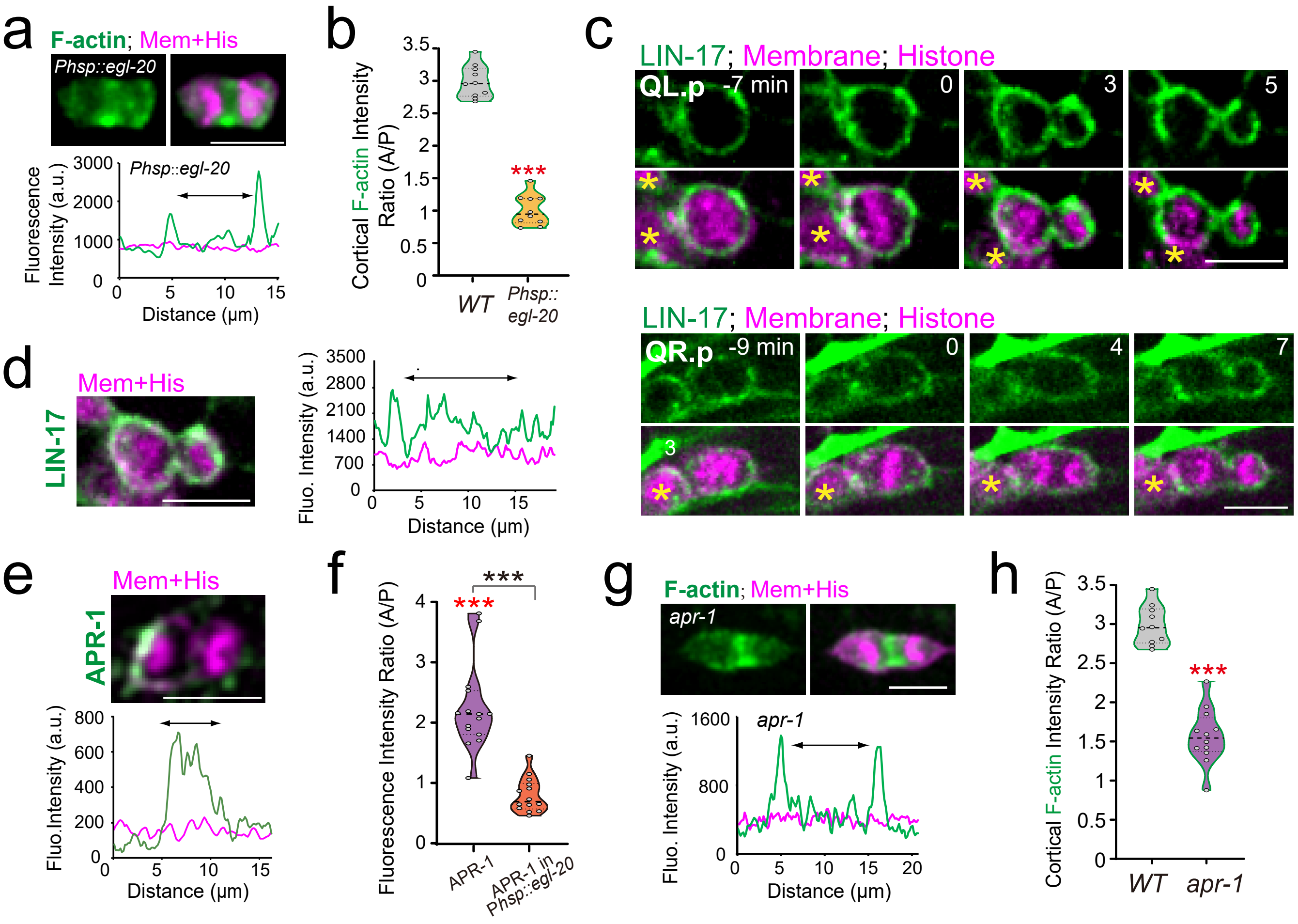

Supplementary Fig. S4. Wnt signaling directs asymmetric actin assembly during QR.p ACD.

(a) Representative images and line scan intensity plots of GFP-tagged moesinABD (F-actin) and mCherry-tagged plasma membrane and histone of cytokinetic QR.p in P*hsp-16.2::egl-20* transgenic worms. **(b)** Violin plots with all data points of the fluorescence intensity ratio of F-actin between the anterior and posterior of the cytokinetic QR.p in WT and P*hsp-16.2::egl-20* transgenic animals (N = 10). **(c)** Fluorescence time-lapse images of GFP-tagged LIN-17 dynamics during QL.p and QR.p cytokinesis. Asterisks indicate neighboring Q cells. **(d)** Representative images and line scan intensity plots of GFP-tagged LIN-17 (green) and Myri-mCherry (magenta) signal around the periphery of QL.p in the inserted images. **(e)** Representative images and line scan intensity plots of GFP-tagged APR-1 (green) and Myri-mCherry (magenta) signal around the periphery of QR.p in the inserted images. **(f)** Violin plots with all data points of the fluorescence intensity ratio of APR-1 between the anterior and posterior of the cytokinetic QR.p in WT (N = 15) and P*hsp-16.2::egl-20* transgenic (N = 14) worms. **(g)** Representative images and line scan intensity plots of GFP-tagged moesinABD (F-actin) and mCherry-tagged plasma membrane and histone of cytokinetic QR.p in *apr-1(ok2970)* mutants. **(h)** Violin plots with all data points of the fluorescence intensity ratio of F-actin between the anterior and posterior of the cytokinetic QR.p in WT (N = 10) and *apr-1(ok2970)* mutant (N = 12) worms. Statistical significance in (b) (f) and (h): ***$p < 0.001$ based on two-tailed Student's t-test. Scale bars in (a), (c)-(e), and (g) 5 µm.

Supplemental Fig. S5

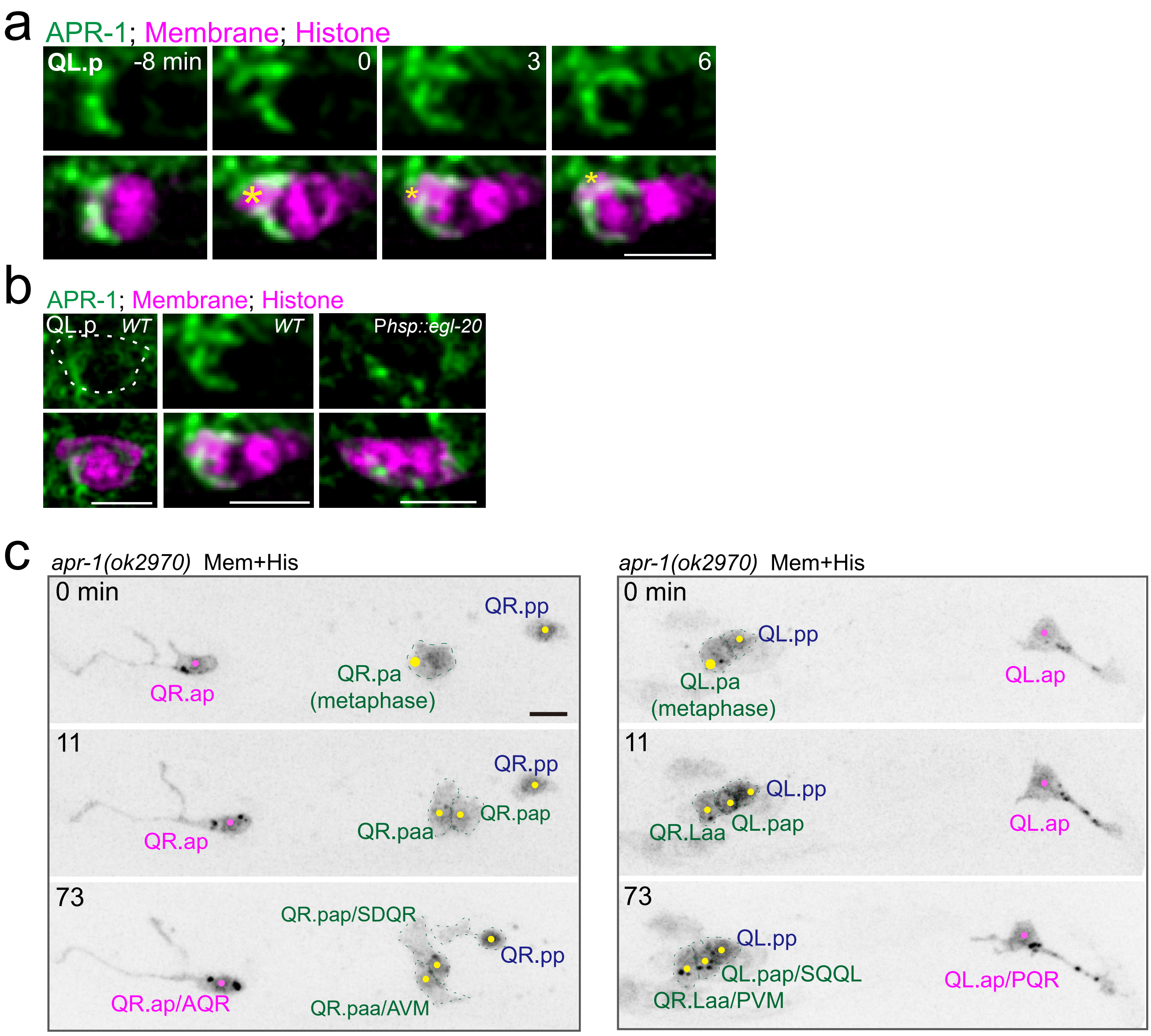

Supplementary Fig. S5. Wnt signaling directs asymmetric actin assembly during QL.p ACD.

(a) Fluorescence time-lapse images of GFP-tagged APR-1 dynamics during QL.p cytokinesis. Asterisks indicate neighboring Q cells. The neighboring epithelial cells (i.e., the hyp7 cell or seam cells) surrounding Q cells have high fluorescence signal from the APR-1::GFP knock-in reporter. **(b)** Representative images of GFP-tagged APR-1, mCherry-tagged plasma membrane, and histone in interphase (left panel: WT) and cytokinetic (middle panel: WT; and right panel: in P*hsp-16.2::egl-20* transgenic background) QL.p. The neighboring epithelial cells (i.e., the hyp7 cell or seam cells) surrounding Q cells have high fluorescence signal from the APR-1::GFP knock-in reporter. **(c)** Fluorescence time-lapse images of the Q.pa and Q.pp fates (QR lineage-AVM and SDQR; QL lineage-PVM and SDQL) in *apr-1(ok2970)* animals. Green dotted lines show the cell periphery. Yellow dots indicate nuclei. The Q.p lineage cell identities are denoted adjacent to the cells. Scale bars in (a)-(c), 5 µm.

Supplementary Videos:

Supplementary Video S1. Dynamic distribution of NMY-2 (NMY-2::GFP) during QR.p asymmetric cell division.

Fluorescence time-lapse images of NMY-2::GFP dynamics during QR.p asymmetric cell division. Frames were taken every 30 seconds. The display rate is 7 frames per second.

Supplementary Video S2. Dynamic distribution of NMY-2 (NMY-2::GFP) during QL.p asymmetric cell division.

Fluorescence time-lapse images of NMY-2::GFP dynamics during QL.p asymmetric cell division. Frames were taken every 30 seconds. The display rate is 7 frames per second.

Supplementary Video S3. Asymmetric plasma membrane expansion.

Fluorescence time-lapse movies of GFP-tagged CMD-1 (Centrosomes) and mCherry-tagged plasma membrane and histones of QR.p during cytokinesis. Frames were taken every 30 seconds. The display rate is 7 frames per second.

Supplementary Video S4. Dynamic distribution of F-actin (GFP::moesinABD) during QR.p asymmetric cell division.

Fluorescence time-lapse images of GFP::moesinABD (F-actin) dynamics during QR.p asymmetric cell division. Frames were taken every 90 seconds. The display rate is 7 frames per second.

Supplementary Video S5. Dynamic distribution of F-actin (GFP::moesinABD) during QL.p asymmetric cell division.

Fluorescence time-lapse images of GFP::moesinABD (F-actin) dynamics during QL.p asymmetric cell division. Frames were taken every 30 seconds. The display rate is 7 frames per second.

Supplementary Video S6. Dynamic distribution of ARX-2 (ARX-2::GFP) during QR.p asymmetric cell division.

Fluorescence time-lapse images of ARX-2::GFP dynamics during QR.p asymmetric cell division. Frames were taken every 30 seconds. The display rate is 7 frames per second.

Supplementary Video S7. Dynamic distribution of WVE-1 (GFP::WVE-1) during QR.p asymmetric cell division.

Fluorescence time-lapse images of GFP::WVE-1 dynamics during QR.p asymmetric cell division. Frames were taken every 30 seconds. The display rate is 7 frames per second.

Supplementary Video S8. Dynamic distribution of WSP-1 (GFP::WSP-1) during Q.p asymmetric cell division.

Fluorescence time-lapse images of GFP::WSP-1 dynamics during QR.p asymmetric cell division. Frames were taken every 30 seconds. The display rate is 7 frames per second.

Supplementary Video S9. Symmetric cell division in *wve-1* conditional knockout mutants.

Fluorescence time-lapse images of GFP::moesinABD during QR.p cytokinesis in *wve-1* conditional knockout. Frames were taken every 30 seconds. The display rate is 7 frames per second.

Supplementary Video S10. Apoptosis of QR.pp cell from asymmetric QR.p cell division.

Fluorescence time-lapse images of the QR.a and QR.p lineage developments in WT animals. Frames were taken every 2 minutes. The display rate is 15 frames per second.

Supplementary Video S11. Survival of QR.pp in *wve-1* conditional knockout mutants.

Fluorescence time-lapse images of the QR.a and QR.p lineage developments in *wve-1* conditional knockout animals. Frames were taken every 2 minutes. The display rate is 15 frames per second.

Supplementary Video S12. Symmetric cell division in *PH::VCA::GFP* overexpression transgenic animals.

Fluorescence time-lapse images of PH::VCA::GFP during QR.p cytokinesis. Frames were taken every 30 seconds. The display rate is 7 frames per second.

Supplementary Video S13. Symmetric cell division in the presence of an ectopic expressed EGL-20.

Fluorescence time-lapse images of GFP::moesinABD during QL.p cytokinesis in P*hsp-16.2::egl-20* transgenic background. Frames were taken every 30 seconds. The display rate is 7 frames per second.

Supplemental Tables

Supplemental Table S1. Plasmids and Primers Used in this Study

Plasmid Name	Primer 5’	Primer 3’
P*egl-17::PH::gfp::VCA*	CCCGAAATGTGAGCTATGGTGAACGATATTCTATTACATGCTGAACG	TTCTTCTCCTTTACTGTGGGTACTTTTTGGTCTGGCTAATAAATG
P*egl-17::CAAX::gfp::VCA*	GTCAAAGACAAAGTGTGTAATTATGAGTAAAGGAGAAGAACTTTTCACTGG	CACTTTGTCTTTGACTTCTTTTTCTTCTTTTTCATAGCTCACATTTCGGGCACCTGAAAAATC
P*egl-17::PH::VCA*	TGCCCGGGGGATCGGT	CCGATCCCCCGGGCACCCCACTTTGTACAAGAAAGCTGGGTTCTTCTGC
GST-VCA	GGGCCCCTGGGATCCATGGTTATAAGCTCGGCTG	TCGACCCGGGAATTCTTAATCATCCCATTCATCATC
P*hsp-6.2::egl-20*	CTTCAAACTATAATCATGCAATTTTTCATTTGCCTG	GAAGAGTAATTGGACTTATTTGCATGTATGTACTGC
P*hsp-16.2::egl-20(n585)*	TCGAGAAAGCGAGAATAAGTTCAAATTTG	TTCTCGCTTTCTCGAATTGCTTCACGAAC

Supplemental Table S2. *C. elegans* Strains Used in this Study

Strain	Genotype
N2	
GOU60	*casIs22[*P*egl-17::gfp-TEV-S::cmd-1*, P*elg-17::Myri-mcherry*, P*elg-17::mCherry-TEV-S::his-24, pRF4].*
GOU174	*casIs35[*P*gcy-32::mCherry, unc-76(+)] X; zdIs5[*P*mec-4::gfp, lin-15(+)] I.*
GOU1544	*casIs165[*P*egl-17::myri-mCherry*, P*egl-17::mCherry-TEV-S::his-24, unc-76(+)] II.; casIs555[*P*egl-17::gfp::moesin] IV.*
GOU1743	*cas607[arx-2::gfp knock-in] V; casIs165[*P*egl-17::myri-mCherry*, P*egl-17::mCherry-TEV-S::his-24,unc-76(+)] II*
GOU2050	*Cas728[gfp::wve-1a knock-in] I; casIs165[*P*egl-17::myri-mCherry*, P*egl-17::mCherry-TEV-S::his-24, unc-76(+)] II.*
GOU1828	*cas723[gfp::wsp-1a knock-in] IV; casIs165[*P*egl-17::myri-mCherry*, P*egl-17::mCherry-TEV-S::his-24, unc-76(+)] II.*
GOU2070	*casEx5256[*P*egl-17::cas9+*P*U6::arx-1-sg*, P*egl-17::myri-mCherry*, P*egl-17::mCherry-TEV-S::his-24]; casIs35[*P*gcy-32::mCherry, unc-76(+)] X; zdIs5[*P*mec-4::gfp, lin-15(+)] I.*

GOU2071	*casEx5257[Pegl-17::cas9+PU6::arx-2-sg, Pegl-17::myri-mCherry, Pegl-17::mCherry-TEV-S::his-24]; casIs35[Pgcy-32::mCherry, unc-76(+)] X; zdIs5[Pmec-4::gfp, lin-15(+)] I.*
GOU2077	*casEx5247[Pegl-17::cas9+PU6::wve-1-sg, Pegl-17::myri-mCherry, Pegl-17::mCherry-TEV-S::his-24]; casIs35[Pgcy-32::mCherry, unc-76(+)] X; zdIs5[Pmec-4::gfp, lin-15(+)] I.*
GOU1626	*wsp-1(gm324) IV; casIs35[Pgcy-32::mCherry, unc-76(+)] X; zdIs5[Pmec-4::gfp, lin-15(+)] I.*
GOU1630	*wsp-1(gm324) IV; casEx5247[Pegl-17::cas9+PU6::wve-1-sg, Pegl-17::myri-mCherry, Pegl-17::mCherry-TEV-S::his-24]; casIs35[Pgcy-32::mCherry, unc-76(+)] X; zdIs5[Pmec-4::gfp,lin-15(+)] I.*
GOU2078	*casEx5261[Pegl-17::cas9+PU6:abi-1-sg1, Pegl-17::myri-mCherry, Pegl-17::mCherry-TEV-S::his-24]; casIs35[Pgcy-32::mCherry, unc-76(+)] X; zdIs5[Pmec-4::gfp,lin-15(+)] I.*
GOU1865	*casEx5271[Pegl-17::abi-1(Modify-Sg1)::gfp; odr-1::gfp]; casEx5261[Pegl-17::cas9+PU6::abi-1-sg1, Pegl-17::myri-mCherry, Pegl-17::mCherry-TEV-S::his-24]; casIs35[Pgcy-32::mCherry, unc-76(+)] X; zdIs5[Pmec-4::gfp,lin-15(+)] I.*

GOU2963	*casEX2592[*P*egl-17::PH::gfp-TEV-S::VCA*, P*egl-17:: myri-mCherry,* P*egl-17::mCherr::his-24]; casIs35[*P*gcy-32::mCherry, unc-76(+)] X; zdIs5[*P*mec-4::gfp, lin-15(+)] I.*
GOU2964	*casEX2594[*P*egl-17::CAAX::gfp-TEV-S::VCA*, P*egl-17:: myri-mCherry,* P*egl-17::mCherry-TEV-S::his-24]; casIs35[*P*gcy-32::mCherry, unc-76(+)] X; zdIs5[*P*mec-4::gfp, lin-15(+)] I.*
GOU2965	*casEX2593[*P*egl-17::PH::TEV-S::VCA]; casIs165[*P*egl-17:: myri-mCherry*, P*egl-17::mCherry-TEV-S::his-24, unc-76(+)] II; casIs555[*P*egl-17::gfp::moesin] IV.*
GOU2966	*casEX2592[*P*egl-17::PH::gfp-TEV-S::VCA*, P*egl-17:: myri-mCherry,* P*egl-17::mCherry-TEV-S::his-24]*
GOU2080	*casEx5247[*P*egl-17::cas9+*P*U6::wve-1-sg*, P*egl-17:: myri-mCherry,* P*egl-17::mCherry-TEV-S::his-24]; casIs555[*P*egl-17::gfp::moesin] IV.*
GOU995	*lin-17(n671) I; casIs35[*P*gcy-32::mCherry, unc-76(+)] X; zdIs5[*P*mec-4::gfp, lin-15(+)] I.*
GOU3209	*lin-17(n671) I; casIs165[*P*egl-17:: myri-mCherry*, P*egl-17::mCherry-TEV-S::his-24, unc-76(+)] II; casIs555[*P*egl-17::gfp::moesin] IV.*

GOU3473	*mhIs9(lin-17::gfp) I; casIs165[*P*egl-17:: myri-mCherry,* P*egl-17::mCherry-TEV-S::his-24, unc-76(+)] II.*
GOU3292	*cp166[mNG-C1^3xFlag::apr-1] I; casIs165[*P*egl-17:: myri-mCherry,* P*egl-17::mCherry-TEV-S::his-24, unc-76(+)] II.*
GOU3844	*cp166[mNG-C1^3xFlag::apr-1] I; casEx5309(*P*hsp-16.2::egl-20,* P*egl-17:: myri-mCherry,* P*egl-17::mCherry-TEV-S::his-24).*
GOU3518	*apr-1(ok2970)I/hT2[bli-4(e937) let-?(q782) qIs48](I;III); casIs165[*P*egl-17:: myri-mCherry,* P*egl-17::mCherry-TEV-S::his-24, unc-76(+)] II; casIs555[*P*egl-17::gfp::moesin] IV.*
GOU3251	*casEx5309(*P*hsp-16.2::egl-20,* P*egl-17:: myri-mCherry,* P*egl-17::mCherry-TEV-S::his-24) ; casIs555[*P*egl-17::gfp::moesin] IV.*
GOU4103	*casEx5309(*P*hsp-16.2::egl-20,* P*egl-17:: myri-mCherry,* P*egl-17::mCherry-TEV-S::his-24) ; casIs35[*P*gcy-32::mCherry, unc-76(+)] X; zdIs5[*P*mec-4::gfp, lin-15(+)] I.*
GOU4104	*casEx5309(*P*hsp-16.2::egl-20(n585),* P*egl-17:: myri-mCherry,* P*egl-17::mCherry-TEV-S::his-24) ; casIs35[*P*gcy-32::mCherry, unc-76(+)] X; zdIs5[*P*mec-4::gfp, lin-15(+)] I.*
